# Relative Risk of High-Danger Industries in China from 2004 to 2016

**DOI:** 10.3390/ijerph17093017

**Published:** 2020-04-27

**Authors:** Lu Zhang, Yun Luo, Ming Xu, Guantao Wang, Wei Liang, Yang Xiang

**Affiliations:** 1 School of Engineering and Technology, China University of Geosciences (Beijing), Beijing 100083, China; zhanglu22930@163.com (L.Z.); luoyun@cugb.edu.cn (Y.L.); wangtgt@cugb.edu.cn (G.W.); 2 Electric Power Research Institute, State Grid Jiangsu Electric Power Co., Ltd, Nanjing 211103, China; wliang1988@163.com; 3 The First Work Zone of the Second Oil Extraction Plant, Daqing Oilfield of CNPC, Daqing 163000, China; walterxy@163.com

**Keywords:** relative risk, high-danger industry, group risk, individual risk

## Abstract

Accidents in high-danger industries in China occur frequently and cause loss of life, injuries, and economic loss. One of the most important reasons causing severe safety situations is the lack of knowledge on macro industry risk in the practice and academic field in China. In order to solve this problem, the method to calculate group risk and individual risk, concerning risk aversion, is discussed, selected, and improved. The group risks and individual risks of five high-danger industries from 2004 to 2016 are calculated. Relative risk indices between five high-danger industries during the 11^th^ and 12^th^ Five-Year Guideline for National Economic and Social Development (FYP) are determined. The results show that there are some differences between group risk and individual risk of high-danger industries. The reasons for differences and the connections with published interventions are discussed. Through discussion, two different ways to reduce group risk and individual risk are identified, which could help supervisors inspect the actual effectiveness of safety policies, measures, and interventions and choose a better way to reduce risk and ensure work safety in industries.

## 1. Introduction

Recently, severe accidents have occurred and caused many casualties and economic losses in China, such as the Tianjin “8.12” explosion, Longyun “10.20” rockburst, and Xiangshui “3.21” explosion. Most of these severe accidents occurred in “high-danger” industries. “High-danger” industry is a conventional concept in China, referring to an industry that has a higher frequency of accidents, or can lead to severe consequences, or can hardly recover from accidents. “High-danger” industries usually include coal mining, metal and nonmetal mining, hazardous chemicals, firework, construction, and so on. *The Law of the People’s Republic of China on Work Safety* stipulates that enterprises in mining, metal smelting, construction, road transport, and hazardous chemicals ought to set up an administrative organization or appoint full-time administrative personnel for production safety [1]. *The Administrative Measures of Withdrawal and Use of Production Safety Expenses*, published by the Ministry of Finance (MOF) and State Administration of Work Safety (SAWS), stipulate that enterprises in high-danger industries ought to withdraw dedicated funds to improve production safety conditions [2].

To tighten the supervision of work safety, the SAWS, formerly the Ministry of Emergency Management (MEM), set up several sectors to take charge of safety supervision in high-danger industries. A series of regulatory interventions have been taken under the joint efforts of multiple administrative departments, such as safety inspection, technical standards, safety management systems, and occupational safety and health (OSH) activities. However, there were still 34046 deaths and enormous economic losses caused by accidents in 2018 [3]. The situation of work safety production in China is still serious. There are a lot of reasons leading to this situation. One of the most important reasons is that decision-makers put more attention on “danger” rather than “risk” because the popularization of the concept of “risk” started late in China. The first Chinese government document which inspects safety risk in the enterprise was published by SAWS in 2014 [4]. This document only contains short regulations on identifying hazards, consequences, prevention, and emergency measures inside enterprises. The government did not truly realize the importance of risk control in production safety until 2016, when “strengthening safety risk control in enterprises” was put forward by China’s highest authority [5]. Only then did safety supervisors at governmental agencies start to strongly carry out supervision involving enterprise safety risk management. However, many safety supervisors are still confused with the differences between “danger” and “risk” and mixing up their meanings [6].

“Danger” and “risk” are two words that are both relative and different from each other [7,8]. “Danger” is usually used to describe “hazard”, which is a source that may cause harm to an asset [9]. “High-danger” is not equal to “high risk” [10]. Risk involves the likelihood and/or consequences of an accident (or a hazardous event) [11]. However, the concept of “risk” was not introduced into China’s safety research field until the 1990s [12,13,14]. Most of the earlier researches focused on the risk of equipment and system combined with the equipment. Nowadays, relevant studies aimed to evaluate risk considering the factors of equipment, human beings, environment, and management inside the high-danger enterprise at the micro level [15,16,17,18,19,20]. However, few studies have evaluated the risk of the whole industry at the macro level. International Labour Organization (ILO) published a national profile report of China in 2012, in which the risks between industries were compared by using deaths per 100,000 workers as the indicator [21]. The annual report of the health and safety executive (HSE) also compared incidence rates (per 100,000 employees) of fatal injuries across the EU in 2015 [22]. In this context, the risk at the macro level refers to the risk of accidents in general in the whole industry. From the view of research subjects, this work does not consider a certain piece of equipment nor a particular kind of harm as the research subject. Instead, it considers the whole industry as a certain “macro hazard” and evaluates its risk, which is different from the meaning at a micro-level. In summary, this research on the risk of a high-danger industry is expected to achieve two goals. One is to improve a method that could calculate the risk of a high-danger industry, to fit in China’s safety environment and conditions nowadays. It could not only be used to describe the risks of high-danger industries in China and the changes in risks these years but also be used to compare the relative risk between these high-danger industries. The other goal is to explore and reveal the connections between the changes in risks and China’s safety situations as well as published policies and measures. In this sense, safety policymakers and supervisors in government could use it as a tool to inspect the effectiveness of interventions.

Risk assessment in safety areas often focuses on accidents that cause consequences, such as fatalities, injuries, economic losses, and environmental pollution. Because sometimes it is difficult to quantitatively evaluate the level of injury, indirect economic losses, and environmental pollution, only the risk involved in human life in fatal accidents are discussed in this article. There is general agreement that risk should at least be judged from two points of view, individual risk and group risk (societal risk) [23]. Individual risk is seen as “the frequency at which an individual may be expected to sustain” harm from specified hazards [24]. Individual risk emphasizes how much danger an individual may face while engaging in a certain activity. It relates to the direct and indirect personal benefits against risk. Group risk is seen as “the relationship between frequency and the number of people” suffering harm in a given population from specified hazards [24]. It emphasizes how dangerous a certain activity is for all people engaged in it. Individual risk and group risk are two different presentations of the same combination of incident frequency and consequences, and both of these measures may be of importance in assessing the benefits of risk reduction measures [25]. Besides, the individual risk could not completely describe situations where a single accident could kill or injure a large number of people [26]. The most significant difference between them is reflected in the fierce reaction from society, which is called societal “risk aversion” [9]. Societal risk aversion is a critical factor considered by policymakers and safety supervisors at government agencies while making decisions and taking interventions in China, especially when the law stipulates that “the main persons in charge of local party committees and governments at all levels are the first persons responsible for production safety” [27]. It may result in safety supervisors paying more attention to the industries that usually cause major accidents and ignoring the industries that have higher individual risk, in which workers may suffer more harm and danger. Therefore, in this article, we decided to evaluate the risk of the high-danger industry considering both group risk and individual risk, beginning with group risk, which could reflect the societal risk aversion. Besides, it needs to emphasize that individual risk discussed here is assumed to be a kind of average risk, and everyone in one specified industry is assumed to have the same level of risk.

This article is organized as follows. In Section 2, we reviewed the methods of group risk and individual risk, discussed the availability, and reported the calculation idea. We also developed the calculation method to compare the relative risk of high-danger industries. In Section 3, the calculation results were shown in detail. The reasons for changes in risks and the relations with published interventions were discussed. Section 4 concluded this work and described the values and instructive meanings for other countries or regions around the world.

## 2. Theory and Methodology

### 2.1. Risk Indicator

The risk indicators used widely to evaluate group risk were the potential loss of life (PLL), the fatal accident rate (FAR), the number of deaths per million (DPM), the FN-curve, and so on. However, all of them have their limitations on achieving the goals mentioned above. The PLL evaluates the expected annual deaths in a specified group or area, but it cannot distinguish the “societal risk aversion” [28]. The same problem exists while using the calculation of DPM. The FAR indicates the number of deaths of people exposed to danger for a certain period [29]. However, it needs the number of average work hours, which are difficult to acquire in China. There are few statistical working hours of high-danger industries in either Chinese government documents or any related researches. *China Labour Statistical Yearbook* only counts working hours by sector, according to the Chinese national standard [30]. The FN-curve draws the frequencies of accidents with N or more deaths [31]. With the FN-curve and FN standard curve, the area of “as low as reasonably practicable” (ALARP) can be divided. However, the criterion based on FN-curves may lead to unreasonable decisions and “does not always come up with the same judgment for the same safety situation” [32].

Vrijling inspected various measures like the potential loss of life (PLL), the area under the FN-curve, and the risk integral and considered that all of them have 2 familiar statistical moments, which were the expected value of the number of deaths and the standard deviation [23]. Thus, the linear combination of the expected value and standard deviation was proposed as the risk aversion measure of group risk. It seems reasonable and feasible that if we have enough available and detailed accident data, in which the most crucial data are the number of accidents involving exactly per number of deaths. However, these kinds of data in high-danger industries could not be found in China. Instead, only the total number of fatal accidents and deaths within each certain scale could be acquired. The Chinese government divided all the accidents into 4 classes of severity scale, as shown in Table 1 [33].

In this case, the expected value of the number of deaths and the standard deviation cannot be calculated because the numbers of accidents involving exactly per number of deaths are unknown. Thus, we decide to go back to the traditional formula of PLL and find a way to solve this problem. If all the people in a specified group have the same individual risk per annum and *n* represents the total number of people working in an industry, then the traditional formula of PLL to evaluate the specified group is as follows:

(1) PLL=n·IRPA

in which

(2) $$IRPA=\frac{Fatal\;deaths\;caused\;by\;accidents}{Total\;number\;of\;people\;affected}$$

Because everyone working in the industry is assumed to have the same level of risk, here we can consider *n* in Equation (1) equal to the total number of people affected in Equation (2).

Furthermore, the PLL can be denoted by considering various types of accidents one by one. The formula related to accidents of type *i* can be described as follows [34]:

(3) $$PLL_{i}=n_{i}\cdot \lambda_{i}\cdot p_{i}$$

where *λ_i_* denotes the frequency of accidents of type *i* per year, *n_i_* denotes the number of persons affected by accidents of type *i*, and *p_i_* denotes the probability that an average person will be killed if an accident of type *i* occurs. Here we use the frequency to estimate the probability *p_i_*, and the historical fatal accident data could be used to acquire it. Hence, the value of *p_i_* could be denoted by the ratio of the death number in accidents of type *i* to the total number of people affected. Thus, the formula of total PLL for all types of accidents is as follows:

(4) $$PLL=\overset{m}{\underset{i=1}{\sum }}PLL_{i}$$

However, there are still two problems with using these equations to evaluate the risk of high-danger industries in China. First, as stated above, these equations cannot reflect “societal risk aversion”. To solve this problem, the solution method named the “aversion multiplier” is designed, which places greater emphasis on the number of deaths [35,36]. Second, there are few data about accident types instead of accident scales in statistic databases in China. We replace the types of accidents with the scales in Equations (3) and (4). Based on the above, the equations to evaluate group risk *R_G_* of high-danger industries can be defined as follows:

(5) $$R_{i}=PLL_{i}=n\cdot \frac{Q_{i}\cdot \frac{D_{i}}{n}\cdot b_{i}}{Y}=\frac{Q_{i}\cdot D_{i}\cdot b_{i}}{Y}\;$$

(6) $$R_{G}=\overset{4}{\underset{i=1}{\sum }}R_{i}\;$$

where *i* denotes the scale standard of accident for *i*=1, 2, …, 4, *n* denotes the total number of employees in an industry, *Y* denotes the number of years counted, *Q_i_* denotes the number of accidents of scale *i*, *D_i_* denotes the total number of deaths caused by accidents of scale *i*, and *b_i_* denotes the “aversion multiplier” index. Relative research indicates that it is appropriate that the value of *b* is in the range of 1 to 2 [37]. Because the scales of accidents are divided into 4 classes, 4 values of *b* are selected from 1 to 2 to reflect the government’s risk aversion attitude for accident death numbers. Noted that the lower limit of death numbers of each scale, 1, 3, 10, 30, has the multiple relationships of 3 approximately, and it could be understood as “risk neutral” when *b* = 1. Hence, the aversion multiplier *b_1_* of an ordinary accident is set to 1, and the next value of *b_2_* could be tentatively set to 1.3, representing the increasing attention about 30%. And so on, the value of *b_i_* could be determined as {1,1.3,1.6,1.9}, while *i* = {1,2,3,4} in this article.

As the formula of group risk is determined, individual risk *R_I_* can be acquired as follows:

(7) $$R_{I}=\frac{R_{G}}{n}\;$$

where *n* denotes the total number of employees in a high-danger industry. The unit of *n* is one hundred thousand people, according to Chinese statistical habits.

After the group risk *R_G_* and individual risk *R_I_* of high-danger industry are determined, we could use 2 tools to directly display and compare the differences between them. The first tool is the percent stacked bar. The percent stacked bar could be used to display the relative proportion of group risk or individual risk of each high-danger industry to the whole. The value of the proportion of each high-danger industry could be acquired by dividing the risk of each high-danger industry by the sum of the risks of all the industries year by year. The second tool is the relative risk index. Relative risk index is designed to quantitatively compare the relative risk between each high-danger industry. It needs a comparable variable, for example, persons. In this sense, it could tell us how many times the risk faced by each person working in one high-danger industry is compared to the risk in another. Therefore, the relative risk index could only be used for comparing individual risk. The first step is to calculate the individual risk of each high-danger industry in 1 year or the average risk value in several years. Next, using coal mining and construction as an example, the relative risk index between these 2 industries could be defined as follows:

(8) $$R_{coal\;mining-construction}^{*}=\frac{\bar{R_{I\left(coal\;ming\right)}}}{\bar{R_{I\left(construction\right)}}}$$

where $R_{coal\;mining-construction}^{*}$ represents the construction relative individual risk of coal mining; $\bar{R_{I\left(coal\;ming\right)}}$ represents the average individual risk of coal mining; and $\bar{R_{I\left(construction\right)}}$ represents the average individual risk of construction. In the same way, other relative risk indices between high-danger industries can be acquired.

### 2.2. Data Collection

The data to calculate are collected from all over China. *China’s Work Safety Yearbook* (CWSY) is organized by the SAWS and written by the administrative sectors in charge of production safety from all the provinces, autonomous regions, municipalities, and departments every year. This book records nationwide statistical data on industrial accidents and occupational diseases, including the number of accidents and casualties by scale, sector in detail, region, type, the status of registration, and so on. According to the statistical data provided by CWSY, we chose coal mining, metal and nonmetal mining, hazardous chemicals, firework, and construction as representations of high-danger industries. The data of hazardous chemicals and firework were independent because they were in charge of different sectors under the SAWS and counted separately. The number of fatal accidents and deaths of different scales in these 5 high-danger industries from 2004 to 2016 was collected in this work [38,39,40,41,42,43,44,45,46,47,48,49,50]. It should be noted that accidents, which caused only injuries but no deaths, were not counted in CSWY. The total number of employees in high-danger industries from 2004 to 2016 were collected from the *China Industry Statistical Yearbook* (CISY) and *China Labour Statistical Yearbook* (CLSY) [51,52]. To evaluate the relative risk index between industries, we chose 2 typical periods, 2006–2010 and 2011–2015, because China uses the “Five-Year Guideline for National Economic and Social Development” (FYP) as the most important policy orientation and action guidance of all fields and the 11^th^ FYP is from 2006 to 2010 as well as the 12^th^ FYP from 2011 to 2015 [53,54,55,56].

## 3. Results and Discussion

### 3.1. Frequencies and Consequences

Risk evaluation of the high-danger industry was based on different scales of fatal accidents in this article. Therefore, the frequencies of different scales of accidents should be noted. There were 66,105 fatal accidents causing 90,886 deaths in the five high-danger industries in China from 2004 to 2016, as reported by CWSY. Table 2 shows the numbers and proportions of ordinary accidents, major accidents, serious accidents, and special major accidents in the five high-danger industries from 2004 to 2016. The proportions of major accidents in hazardous chemicals and firework were much larger than those in coal mining, metal and nonmetal mining, and construction.

**Figure 1:**
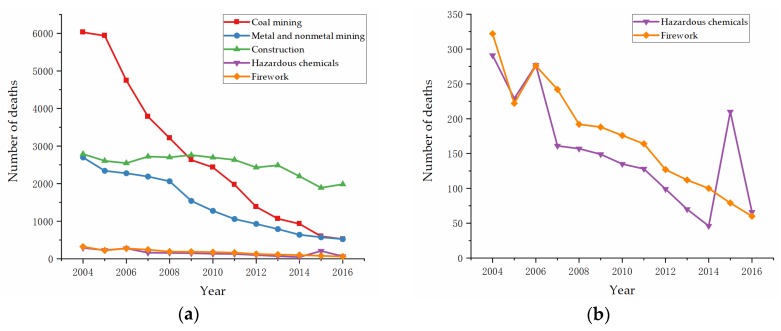
Numbers of deaths caused by accidents in high-danger industries in China: (**a**) Coal mining, metal and nonmetal mining, construction, hazardous chemicals, and firework; (**b**) hazardous chemicals and firework.

The number of deaths caused by accidents in high-danger industries are shown in Figure 1a,b. Because the curves of hazardous chemicals and firework were too close to be distinguished in Figure 1a, they are separately shown in Figure 1b. Figure 1a,b show a declining trend in the numbers of deaths in coal mining, metal and nonmetal mining, hazardous chemicals, and fireworks overall. The Tianjin “8.12” explosion occurred in 2015, which caused 165 deaths, 8 missing persons, 798 injuries, and 6.866 billion yuan (almost 1.068 billion dollars) direct economic loss, which was why there was a high point in hazardous chemicals in 2015 in Figure 1b. Moreover, the number of casualties in construction declined from 2789 deaths in 2004 to 1984 deaths in 2016. In 2009, construction exceeded coal mining and became the industry with the highest number of fatalities for the first time.

### 3.2. Risk Evaluation

#### 3.2.1. Group Risk

**Figure 2:**
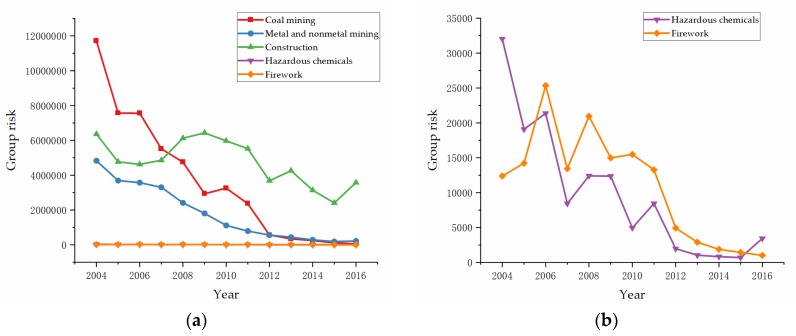
Group risk of high-danger industries: (**a**) Coal mining, metal and nonmetal mining, construction, hazardous chemicals, and firework; (**b**) hazardous chemicals and firework.

Risk evaluation was based on the data of the five high-danger industries collected by CWSY from 2004 to 2016. Equations (5) and (6) were used to evaluate the group risk of each high-danger industry. Since we used the overall data for each year and the number of deaths per accident cannot be collected, there exists the limitation of data confidence, which will be discussed in the following discussion section. The results (Figure 2a,b) showed that all the group risk of five high-danger industries decreased in total. The industry of coal mining had the highest risk in 2004, and construction has been the industry with the highest risk since 2008. Compared to construction, there was only a small difference between coal mining, metal and nonmetal mining, hazardous chemicals, and firework from 2012 to 2016.

**Figure 3:**
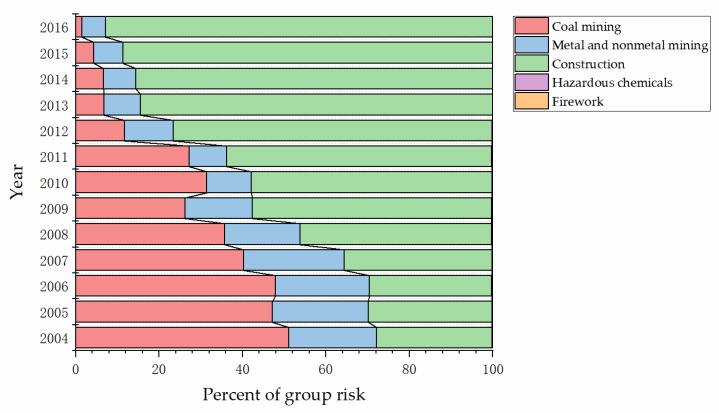
Percent stacked bar of group risk of each high-danger industry to the whole.

The percent stacked bar was used to display the relative relationship of group risk to the whole for each high-danger industry. The results are shown in Figure 3, which directly reveals the proportions of the five high-danger industries and the rapid increase in the proportion of construction industry.

#### 3.2.2. Individual Risk

**Figure 4:**
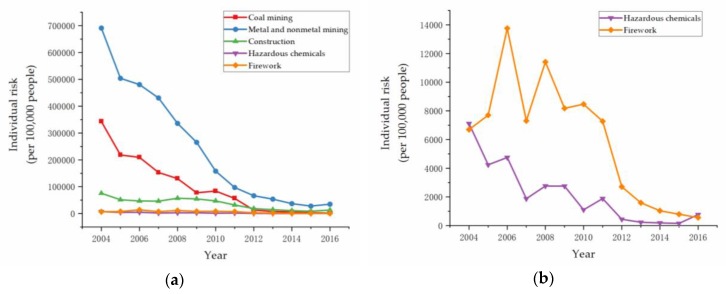
Individual risk of high-danger industries: (**a**) Coal mining, metal and nonmetal mining, construction, hazardous chemicals, and firework; (**b**) hazardous chemicals, and firework.

Individual risk provided a way to compare how much danger one person faced in one industry compared to another. According to Equation (7) and data collected from CWSY, CISY, and CLSY, the individual risk of each high-danger industry was calculated, as shown in Figure 4a,b.

The results showed that there was an overall downward trend of individual risks in all five industries. Different from group risk, the individual risk of metal and nonmetal mining was extremely high in 2004. Moreover, the individual risk of construction was in third place at the same time. In Figure 4b, the individual risk of firework was much higher than that of hazardous chemicals from 2006 to 2011. A similar result was also found in Figure 2b.

**Figure 5:**
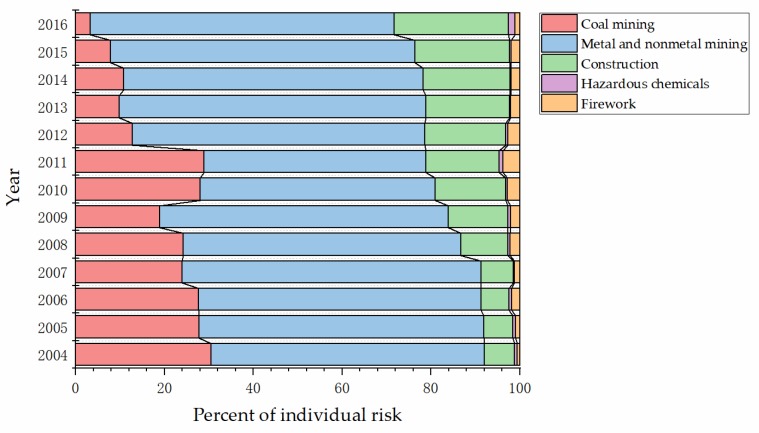
Percent stacked bar of individual risk of each high-danger industry to the whole.

The percent stacked bar was also used to present the relative relationship of individual risk to the whole for every high-danger industry. As shown in Figure 5, the proportion of the individual risk of metal and nonmetal mining always had a proportion of more than half from 2004 to 2016. 

To describe the relationship of individual risk of the high-danger industries more precisely, the relative risk index was used to calculate the proportion between high-danger industries. The results are shown in Table 3 and Table 4. The numbers in Table 3 and Table 4 represent the multiple relationships of the degree of individual risk between any two industries. 

#### 3.2.3. Discussion

**Figure 6:**
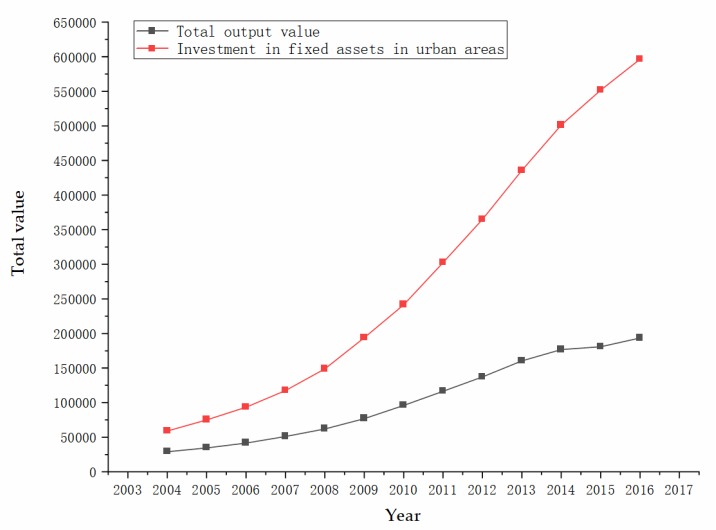
Total output value of construction and investment in fixed assets in urban areas in China.

As mentioned in Section 1, group risk conveys the risk level of all the people engaged, and individual risk conveys the risk level of each person. The same group risk with a different number of employees could make a significant difference in the result of individual risk. Based on the figures and tables above, we can see the differences between group risk and individual risk of industries. We believe both of them could be not only important in assessing the benefits of measures as mentioned above, but also have different reasons for differences and changes. In Figure 2, the changes in group risks between coal mining and construction are evident. China is one of the most important coal-producing countries in the world. There are loads of small coal mines operating under poor production conditions and lacking basic safety protections, which may cause many accidents. The Chinese government has adopted three main policies to change this situation. First, the government accelerated the elimination of outdated coal mines with low production capacity and started to shut down 8000 small coal mines identified by lacking safe production conditions, non-compliance with industrial policy, resource-wasting, and environmental pollution from 2010 [57]. Second, coal mines across the whole country began to merge and reorganize, with the aim to increase coal mine annual production capacity from less than 300,000 tons to more than 800,000 tons per year [58]. Third, six advanced safety systems were demanded to install in every coal mine: Monitoring system, underground personnel location system, emergency refuge system, compressed air self-rescue system, water rescue system, and communication system [59]. As for construction, the most likely explanation for the increase from 2008 is that relevant economic and production activities increased [60]. When the global financial crisis broke out, the Chinese government invested 4 trillion yuan in rescuing the market. As a result, a large amount of hot money poured into real estate markets and infrastructure construction industries, which greatly boosted the construction industry. Figure 6 shows the trend of the total output value of construction and investment in fixed assets in urban areas from 2004 to 2016, reflecting the rapid increase in relevant construction activities since 2008.

The group risk and individual risk of the firework industry suddenly increased in 2006, as shown in Figure 2b and Figure 4b. It may closely relate to the sudden increase in nationwide demand for fireworks. In 2006, the Beijing City government abrogated the regulation that had banned fireworks for 12 years, and more than 160 cities in China followed. This move greatly stimulated the production of fireworks. Besides, firework production in China is a labor-intensive industry. The production scale is generally small, with simple process equipment and low technology. Moreover, illegal productions of fireworks are common in the countryside. These factors led to the high individual risk of fireworks. This situation lasted 6 years and did not reverse until the strong push of the nationwide firework enterprise work safety standardization and construction of the national firework flow management system began in 2011.

In Figure 4, metal and nonmetal mining had the highest individual risk. There are some potential explanations. The first is that there was a very large proportion of small metal and nonmetal mines in China. There were 33,156 (87.6%) small, 2979 (7.9%) medium and 1710 (4.5%) large metal and nonmetal mines by the end of 2017 [61]. Small mines use outdated and unreasonable mining technology, such as the room-and-pillar method and shallow hole blasting [62]. Secondly, the proportion of surface mines were 76.6% [61]. Surface mines mostly excavate limestone and stones for buildings, which are low-value minerals bringing low economic benefits, hence it is more difficult for high-cost mining technology with high-level safety to be applied to these surface mines. In addition, illegal mining had become so widely spread and serious that the government put forward many efforts to end this [63,64].

The reasons for these differences above are comprehensive, combining both the characteristics of the industry itself and the influence of China’s policy orientation. Supervisors at government agencies mostly focus on the industries that cause more deaths in one accident or in each year due to their greater nationwide influence. However, from the perspective of ensuring labor health and occupational safety, it is necessary for supervisors to focus on industries with high individual risks, such as metal and nonmetal mining, and to take immediate regulatory interventions.

Moreover, there are two different ways to control the group risk and individual risk of industry, based on the 13-year trend on risks of high-danger industries and the order of the announcement of policies in China. Group risk is closely related to the production capacity of the whole industry. The optimal way to decrease group risk is to optimize the structure and increase the unit capacity of the industry. Once group risk decreases, the old measures would no longer be useful. Therefore, another way to reduce individual risk and pay more attention to personnel life and health needs to be taken. To achieve this goal, the intrinsic safety of enterprises must be appreciated. Relative interventions and measures should include improving production technology and the reliability of equipment, constructing safety production and supervision systems, complying with safety production standardization, developing a safety culture, and so on. It should be noticed that the safety management system of risk ranking and control in enterprises has been widely promoted as a critical method to prevent accidents these years by the State Council [65]. Especially, in the safety field of metal and nonmetal mining and hazardous chemicals, certain regulations and measures are published, aiming at putting more attention on identification, evaluation, monitoring, reduction, and informatization of safety risk [66,67]. Besides, the nationwide procedure of model city appraisal in safety development has been in place since 2019 [68]. The policy effect of safety risk regulation has a high proportion of the scoring criteria, which aims to stimulate the work of risk control in all enterprises and industries. 

Nevertheless, the limitations of this article should also be pointed out. There are many kinds of consequences caused by accidents, and human life represents merely one part of them. There is a large challenge to unify the dimensions among the loss of human life, economic loss, environmental, and ecological destruction. When it comes to human life and health, the problem to evaluate both deaths and different levels of injuries remains unsolved. Besides, we used the calculation data provided by China’s Work Safety Yearbook (CWSY), China Industry Statistical Yearbook (CISY), and China Labour Statistical Yearbook (CLSY). Although they are the most detailed data resources that could be found in the public, we should be aware of the effect caused by data accuracy. However, data accuracy may be influenced by many factors, such as sampling errors, statistical errors, even local concealed or false declarations on purpose. In this case, this method could be improved by using 95% confidence intervals, only if we could get more detailed information on accidents and deaths. Thus, it faces challenges coming from both methods and data sources in China.

## 4. Conclusions

To evaluate the risk of high-danger industries, we improved a method for calculating both group risk and individual risk of the industry, as well as the relative risk between high-danger industries. The calculation method was used to acquire results with data in the five high-danger industries from 2004 to 2016 in China. Based on the individual risk of high-danger industries, relative risk indices were acquired during the 11^th^ and 12^th^ FYP in China. The group risks and individual risks of five high-danger industries show significant differences both in the contexts of types and timelines. The relative key points and their reasons were analyzed. It is helpful for safety supervisors to identify, at both the group and individual levels, which industry has higher risk and which has less risk. Based on the trends and discussions of Chinese production safety policies, regulations, measures, and interventions, we can choose different ways to reduce the group risk and individual risk of industry.

This work would be helpful for supervisors to check the possible deficiencies in published policies, as well as take further effective measures to safeguard work safety. It could remind us to realize the differences between the habitual perception of “high-danger industry” and the fact of “high-risk industry”. Besides, it could also provide a referential calculation method for countries and regions using similar accident statistical patterns like China, which counts and publishes accident data according to the severity instead of the type of accident. Finally, it should be noted that although the method is based on safety situations and political environment in China, it could also be internationally universal and useful for countries or regions around the world.

## Figures and Tables

**Table 1:** Four scales and standards of accidents in China.

Scale of Accident (*i*)	Standards of Accident Scale
Special major accident (*i*_4_)	Causing more than 30 deaths, more than 100 serious injuries (including acute industrial poisoning, the same below), or more than 1 × 10^8^ yuan in direct economic loss
Serious accident (*i*_3_)	Causing more than 10 and less than 30 deaths, more than 50 and less than 100 serious injuries, or more than 5 × 10^7^ and less than 1 × 10^8^ yuan in direct economic loss
Major accident (*i*_2_)	Causing more than 3 and less than 10 deaths, more than 10 and less than 50 serious injuries, or more than 1 × 10^7^ and less than 5 × 10^7^ yuan in direct economic loss
Ordinary accident (*i*_1_)	Causing less than 3 deaths, less than 10 serious injuries, or less than 1 × 10^7^ yuan in direct economic loss

**Table 2:** Numbers of four scales of accidents in five high-danger industries in China (2004–2016).

Industry	Total Number of Accidents	Number of Ordinary Accidents	Number of Major Accidents	Number of Serious Accidents	Number of Special Major Accidents
Coal mining	20977	19462 (92.78%)	1203 (5.73%)	274 (1.31%)	38 (0.18%)
Metal and nonmetal mining	15302	14787 (96.63%)	491 (3.21%)	21 (0.14%)	3 (0.02%)
Construction	27586	26821 (97.23%)	744 (2.70%)	18 (0.07%)	3 (0.01%)
Hazardous chemicals	1074	937 (87.24%)	128 (11.92%)	8 (0.74%)	1 (0.09%)
Firework	1166	1020 (87.48%)	124 (10.63%)	21 (1.80%)	1 (0.09%)

**Table 3:** Relative risk index between five high-danger industries during the 11^th^ FYP in China.

Relative Risk Index	Hazardous Chemicals	Firework	Construction	Coal Mining	Metal and Nonmetal Mining
**Hazardous chemicals**	1	0.27	0.05	0.02	0.01
**Firework**	3.71	1	0.20	0.08	0.03
**Construction**	19.00	5.13	1	0.39	0.15
**Coal mining**	49.33	13.31	2.60	1	0.39
**Metal and nonmetal mining**	126.04	34.01	6.63	2.56	1

**Table 4:** Relative risk index between five high-danger industries during the 12^th^ FYP in China.

Relative Risk Index	Hazardous Chemicals	Firework	Construction	Coal Mining	Metal and Nonmetal Mining
**Hazardous chemicals**	1	0.22	0.03	0.03	0.01
**Firework**	4.62	1	0.16	0.16	0.05
**Construction**	29.07	6.29	1	0.98	0.30
**Coal mining**	29.65	6.41	1.02	1	0.30
**Metal and nonmetal mining**	97.31	21.04	3.35	3.28	1
